# Tools and challenges in the use of routine clinical data for antimicrobial resistance surveillance

**DOI:** 10.1038/s44259-025-00105-3

**Published:** 2025-05-09

**Authors:** Kathryn E. Holt, Megan E. Carey, Clare Chandler, James H. Cross, Zoe A. Dyson, Nicholas Furnham, Rebecca E. Glover, Mollie Virgo, Gwenan M. Knight

**Affiliations:** 1 London School of Hygiene & Tropical Medicine, London, WC1E 7HT UK; 2 School of Translational Medicine, Monash University, Melbourne, VIC 3004 Australia; 3 IAVI, Chelsea & Westminster Hospital, London, UK; 4 Wellcome Sanger Institute, Wellcome Genome Campus, Hinxton, UK

**Keywords:** Clinical microbiology, Policy and public health in microbiology, Bacterial infection

## Abstract

Routine clinical microbiology data are widely used for antimicrobial resistance (AMR) surveillance, but data availability and quality vary. In this Perspective, we explore the technical challenges of utilising routine data to inform action at various levels, and summarise emerging open-source technical solutions for hospital-level data collection, aggregation, and sharing. We highlight a need for agreed-upon data standards, and tools that support both facility-level and public health surveillance.

## Introduction

In September 2024, member states at the United Nations General Assembly High-Level Meeting on Antimicrobial Resistance (AMR) reached agreement on a political declaration on AMR^1^. The declaration establishes a target of 10% reduction in deaths associated with bacterial AMR by 2030, predicated on a recent baseline estimate of 4.95 million deaths in 2019^2^. Key strategic steps required to meet this target include improving access to microbiological diagnostics (i.e., identifying the agent of infection and, where possible, testing its susceptibility to potential antimicrobial therapeutics) and AMR surveillance to inform action against AMR.

**Fig. 1:**
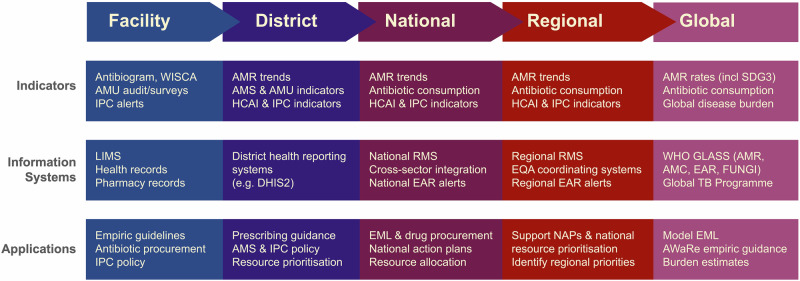
The AMR data journey, from facility-level data collection and utilisation, to informing global policy. Abbreviations: Antimicrobial Resistance (AMR), Antimicrobial stewardship (AMS), Antimicrobial Consumption (AMC), Antimicrobial Usage (AMU), AWaRe (Access, Watch, Reserve), Emerging AMR Reporting (EAR), Essential Medicines List (EML), Global Antimicrobial Surveillance System (GLASS), Healthcare-Associated Infections (HCAI), Infection Prevention and Control (IPC), Laboratory Information Management System (LIMS), National Action Plan (NAP), Reporting and Monitoring System (RMS), SDG (Sustainable Development Goal), Tuberculosis (TB), World Health Organization (WHO), Weighted-incidence syndromic combination antibiogram (WISCA).

**Fig. 2:**
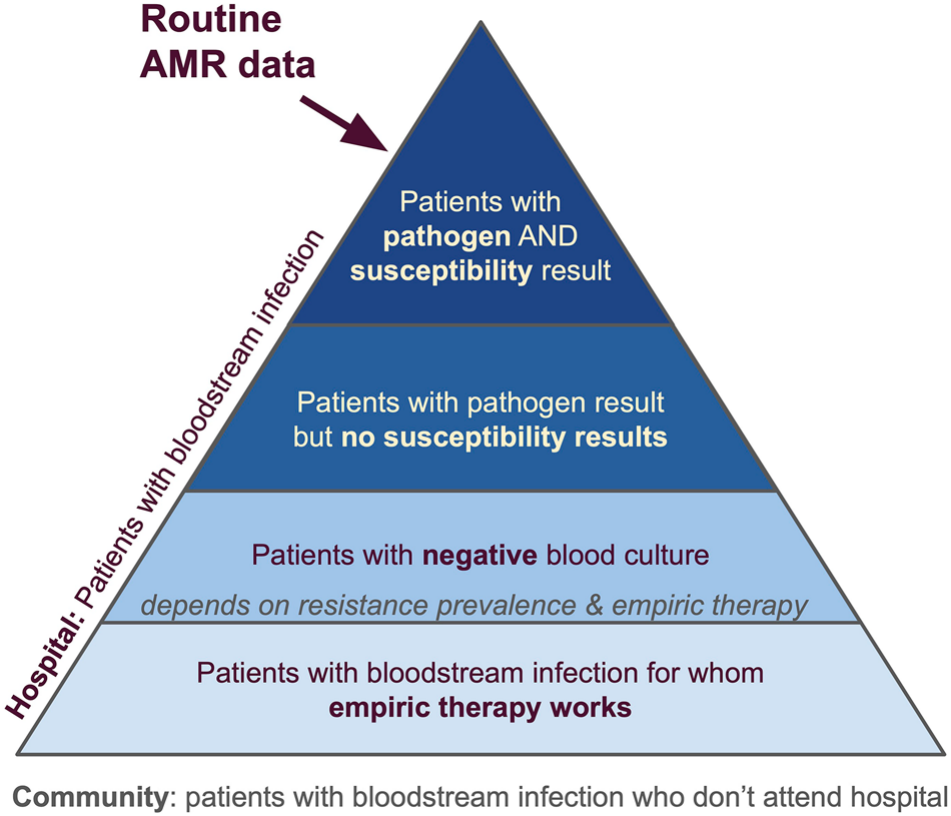
Routine AMR data represent a subset of total infections.

Most data informing AMR burden estimates rely on routine microbiological testing. In some settings, primarily hospitals in high-income countries, microbiological diagnostics are a core component of individual patient care and infection control. Where available, this routine clinical information is a rich data source for AMR surveillance^3^. When aggregated, such data can be used to estimate prevalence of resistance to specific antibiotics amongst specific bacteria (bug-drug combinations) at a facility level, reveal dynamic trends that can be highly informative for hospital management to guide local empiric therapy and antibiotic procurement^4^, and support the identification of infection control issues should they arise (Fig. 1). Further aggregating facility-level data at district or national level can then give a picture of AMR prevalence and emergence, revealing trends at a higher level across time and space. The value of routine microbiology data for informing AMR action can be further enhanced by linkage with clinical data (i.e., from hospital records) and pharmacy data on antimicrobial use. These granular and aggregated data can inform national surveillance, and feed into empiric antibiotic guidance tailored to patient subgroups (e.g. via weighted-incidence syndromic combination antibiograms (WISCA)^4,5^), antibiotic procurement, and AMR National Action Plans (Fig. 1). National data can also be transmitted to international authorities at the supranational region level (e.g. the European Centre for Disease Prevention and Control (ECDC) or Pan American Health Organization), and the Global Antimicrobial Surveillance System (GLASS), managed by the World Health Organization^6^, illustrating regional trends, tracking progress toward target reduction, and informing international actions (Fig. 1). Blood culture data from hospitals are considered particularly useful for surveillance across settings, as they can be reasonably assumed to represent severe infections without requiring complex diagnostic criteria that are difficult to standardise^7–9^. However these data naturally reflect the more severe ‘tip of the iceberg’ of infections, enriched for those that did not respond to empiric therapy (Fig. 2).

Whilst routine clinical microbiology data provide a potentially rich source of information for AMR surveillance, data availability varies widely between countries and clinical settings^10^, and their use for this secondary purpose can prove challenging from a logistical, ethical and legal perspective. Tackling these challenges, and exploring the limitations and potential of these sources of data, is vital as we enter a time of target-based AMR control and further efforts to quantify and reduce AMR burden. In this Perspective we explore some of the technical challenges of data curation, and focus particularly on emerging technical solutions for data curation and linkage at the hospital level and for data aggregation and sharing at different scales. While multiple other challenges also impact data used for surveillance, such as the representativeness of the populations tested or the ability to develop effective policy or practice responses to findings, our focus here is on the curation of data once a patient’s sample is within the system until it is presented as a datapoint in surveillance outputs, rather than the broader challenges associated with generating microbiological data which have been addressed elsewhere^11^.

## Hospital-level AMR data collection, validation, and transmission

Using routine hospital data for AMR surveillance ideally requires synthesis of data from three key sources: microbiology data (what is the agent of infection, and what drugs is it resistant or susceptible to?); patient data (demographics including age, sex, occupation, and where they live); and clinical data (such as symptoms, severity, co-morbidities, vaccination status, admission pathway, outcome, and antimicrobial exposure, including which drug/s the patient was treated with, timing/duration, and dose).

These different types of routine surveillance data are typically collected and stored in different systems – microbiology data in a laboratory information management system (LIMS; which may be provided or populated by an external service provider), patient and clinical information in healthcare records (e.g. paper-based records or an electronic healthcare records system, EHR), and antimicrobial use data in a pharmacy database. Some aspects of microbiology and pharmacy data might be reported into the healthcare record, but the mechanisms and level of detail vary. Thus, gathering data on AMR, for local facility use and onward transmission to national or regional data collectors tends to require a specific step of data linkage. In settings where records are paper-based (including most low- and middle-income countries (LMICs)) there is an additional step of digitisation needed.

Incorporating facility-level LIMS and EHR represents a practical solution for leveraging routine clinical data to enhance local AMR surveillance and support quality improvement initiatives within a healthcare facility. By combining these systems, facilities can more effectively manage and utilise diagnostic laboratory and clinical care data, enabling laboratory staff to contribute to patient management and clinical decision-making. For instance, laboratory insights, such as AMR trends, can directly inform local treatment decisions when data are effectively linked to clinical outcomes^4^. These insights are valuable for helping refine treatment protocols and improving infection management at the local level, where the data’s context is best understood. Digitising laboratory and clinical data can therefore improve local patient care, as well as enhancing the quality and reliability of data for onward transmission to regional or national surveillance systems, aligning local data utility with broader public health objectives.

Transmission of data outside the facility and aggregation of data from multiple facilities requires several additional considerations, primarily the ability to easily extract data in suitable formats that are fit-for-purpose in terms of key issues such as protection of patient privacy, and encoding of data according to common standards. It is also vital to communicate information on sampling frameworks to ensure that any bias in the patient population is accounted for along with the data. Depending on the data pathway and its governance, data may need to be de-identified before being transmitted out of the facility. In such cases samples it is important that samples are de-duplicated before being transmitted, as including multiple samples per patient can have a significant impact on AMR prevalence estimates^12^. A common strategy is to include the first isolate per pathogen per patient per specimen type in the given surveillance period (e.g. for reporting to WHO GLASS). Notably this approach cannot account for patients who have been sampled at multiple facilities; such cases can only be detected and de-duplicated by data aggregators if facilities can share identifiable data through appropriately protected systems (e.g. this approach is used by the United Kingdom Health Security Agency, in accordance with relevant data protection requirements).

Data standards are an important consideration when sharing and aggregating AMR data, and can be built into LIMS and EHR systems to facilitate export of data suitable for external transmission, or used to post-process received data before aggregation. For laboratory data, there needs to be a standardised ontology for key variables such as the species name, specimen types, and drug names; and the recording and interpretation of laboratory assay data must be considered. Several different assays can be used to assess antimicrobial susceptibility of bacteria, e.g., microbroth dilution or E-test to assess minimum inhibitory concentration, or disk diffusion assays. There are different standards by which these assay measures can be interpreted, which change over time, into categories of susceptible vs intermediate vs resistant (S/I/R), the most common being those published by the European Committee on Antimicrobial Susceptibility Testing (EUCAST) or Clinical & Laboratory Standards Institute (CLSI). When transmitting AMR data, knowing which assay was used and how the output was interpreted is important. In general, it is best to transmit the raw assay measures, together with information on methods and instrumentation used, so that they can be reinterpreted as our understanding of the clinical relevance of laboratory assays advances^13^. Variable encoding is also complicated for patient and clinical data. Even something as apparently simple as recording ‘age’ has complexities – one might simply record age in years; however, this is inadequate for patients under 1 as it is important to distinguish neonates from infants (and additional granularity may also be desired), and standards need to be in place for unknown or uncertain ages. The need for granularity of age in some contexts must be balanced against the potential risks of data triangulation being used to identify individual patients; standardised age groups appropriate to the specific context and disease syndrome can be helpful here^14^. There are various data standards for clinical data, and it is important to distinguish raw variables from clinical findings (e.g. ‘specimen: sputum’ vs ‘diagnosis: pneumonia’) and to use consistent clinical definitions such as International Classification of Diseases (ICD-10) codes. For infections in hospital settings, key considerations include definitions of specific syndromes (needed to define weighted-incidence syndromic combination antibiograms (WISCAs))^4,5^; definitions of healthcare-associated infection (used to inform infection prevention^15^); and definitions of outcome (e.g. deaths in hospital vs deaths after discharge, often used to assess burden of AMR^2^).

It is also important to consider which elements of data are transmitted from a facility to national reference centres, and from there to international databases. Protecting patient privacy, and vulnerable communities, is essential, and dedicated resources may be needed to ensure appropriate data quality and governance for different purposes. The data is most valuable to those for whom it is most proximal and that is where the most granularity is needed, it is also where the biases and caveats are best understood. However, the risks and benefits of data sharing at different levels need to be considered and clarified at each step of the data journey.

## AMR data tools: from the hospital to the world

There is a large ecosystem of tools relevant to the collection, analysis, and sharing of AMR data in the context of routine clinical services in hospital settings. Rather than providing an exhaustive list, here we outline the key elements required and highlight examples of free (and mostly open-source) software tools that have been developed to address particular steps in the data journey from hospital laboratories to national and international surveillance systems.

A Laboratory Information Management System (LIMS) is software designed to support data collection and storage within a laboratory, tracking specimens as they move through workflows, recording and linking results from different assays through integration with laboratory instruments, and interfacing with other databases and information systems such as patient records. Clinical microbiology laboratories have specific requirements^16^, including the need to track multiple specimens from the same patient (e.g. a blood sample and a urine sample); multiple culture media and multiple microbial isolates derived from those specimens; and multiple assay results for each isolated pathogen (species identification, susceptibility to a panel of antimicrobials).

Whilst many commercial LIMS are available, the Surveillance and Epidemiology of Drug-resistant Infections Consortium (SEDRIC) in 2019 identified the need for a free and open-source, microbiology-focused LIMS suitable for deployment in LMICs. SEDRI-LIMS was subsequently developed and is now available at a range of scales from single workstations to local servers and cloud-based setups, making it suitable for diverse laboratory environments; source code is due to be released in April 2025 (https://www.sedrilims.com/). In addition to the requisite features outlined above, SEDRI-LIMS supports sample barcoding, interpretation of susceptibility test results into S/I/R categories, and data export (to WHONET format described below, with other formats planned). This complements the SILAB for Africa LIMS developed to support veterinary laboratories in Africa^17^.

WHONET is a free Windows-based application designed to support clinical microbiology laboratories with the management, analysis, and reporting of AMR data (https://whonet.org/). It can be populated by data extracted from a LIMS, or by direct entry of sample-level data on species identification and susceptibility test results (including data exported from automated susceptibility testing platforms, via the integrated BacLink tool). The software also supports interpretation into S/I/R categories, facility-level summaries and cluster alerts (e.g. increased AMR in a particular ward, supported by the integrated SaTScan software), and exporting of data in a variety of formats used by public health surveillance programs (including the ‘WHONET’ format which is required for submission of data to WHO GLASS). Training materials, online courses, and webinars are available to help ensure the software is sustainable and empower local and regional stakeholders to support tasks ranging from annual surveillance to real-time decision-making and policy advocacy.

The AutoMated tool for Antimicrobial resistance Surveillance System (AMASS, https://amass.website/) is a free and open-source software package designed to support the use of AMR data at facility level, as well as national and regional surveillance, through linkage of laboratory and clinical data^18^. It is an offline tool that takes as input both AMR data (extracted from a LIMS or WHONET) and electronic health records (in CSV or Excel format), links these records at patient level, and exports de-identified data suitable for transmission to national surveillance networks (CSV format). AMASS also generates facility-level summaries (e.g. bug-drug AMR prevalence, stratified by community vs hospital-acquired), which can be exported as PDF reports or CSV data summary files, and can generate automated reports on AMR and notifiable bacterial diseases for transmission to national authorities^19^. In addition to providing AMR statistics including AMR proportion, AMR frequency and case fatality rate and total number of deaths following AMR bloodstream infection, the software also reports quality indicators like contamination rates and infrequent antibiotic resistant profiles. Future goals include integrating microbiology, hospital admission, and pharmacy data. The utility of AMASS for country-wide AMR surveillance was assessed by the Ministry of Health in Thailand in 127 public hospitals nationwide^20,21^; it is also being deployed in other LMICs^9,22^. Future goals include integrating microbiology, hospital admission, and pharmacy data.

Whilst the above tools have some flexibility to deal with the often messy error-prone and non-standardised data from across different systems, supplementary tools may be needed for more specialised data tasks. One such tool is the AMR R package, which provides a range of statistical tools to standardise and facilitate analysis of complex routinely-collected AMR data^23^. Key features include functions for applying EUCAST or CLSI guidelines to interpret assay results, and tools to correct errors and standardise microorganism and antibiotic nomenclature.

There are several configurations in which the tools outlined above can work together or be used interchangeably, and connect with other tools such as DHIS2 (a general open-source platform for data integration and visualisation, https://dhis2.org/), to support AMR surveillance. For laboratories without a LIMS, WHONET may be used as a primary tool for data entry, analysis, and onward sharing. Those with a LIMS might choose to export data to WHONET or AMASS for analysis and reporting, and this decision may be influenced by the need to interact with national reference centres and surveillance programs. Notably, each of these tools use different formats to store AMR data (with WHONET emphasising interoperability of formats via their BacLink tool), and implement their own code to interpret susceptibility assay data into S/I/R categories using EUCAST or CLSI guidelines (which need to be digitised, and frequently updated to keep pace with updated guidelines). These areas would benefit from the development of agreed standards to improve interoperability and robustness, reduce the burden of software development and maintenance, and facilitate cross-sector integration of AMR data across the One Health continuum. For example, the One Health AMR Surveillance (OHAMRS) system in Kenya reports collecting data from hospitals via a mix of LIMS (43%), WHONET (19%), and a custom MS-Excel template (38%); and from veterinary laboratories via the SILAB LIMS; which then had to be integrated via DHIS2 to create surveillance dashboards^24^.

## Limitations of routine AMR data for surveillance

Despite the widespread reliance on routine clinical data to survey AMR, there are many limitations. One key issue with use of data collected through routine hospital care, in particular aggregated data and comparisons of data between settings, is that what is ‘routine’ can vary enormously (Fig. 2). In low-income countries microbiological diagnostics may not be available routinely or at all^11^, so there is no routine data to capture for surveillance purposes. The World Health Organization highlights the need to prioritize health system strengthening generally, and access to diagnostics and antibiotics specifically, in order to reduce AMR-associated deaths^1,25^. A secondary benefit of such strengthening could be increased availability of data for surveillance. In high-income countries with nationalised healthcare systems and well-resourced laboratories, all patients with suspected severe infection likely receive a blood culture, and it might be assumed that the culture-positive rate, or the rate at which a culture identifies a pathogen, will be high and the contamination rate will be low. However, which antibiotics are tested for can vary between settings and over time, affecting the apparent prevalence of resistance (i.e., a lack of evidence for a specific resistance in a specific setting may be because that antibiotic is rarely tested, not because resistance is absent)^26^. In contrast, if diagnostic culture is tied to willingness to pay out-of-pocket, or laboratories are under-resourced^27^ or there is a lack of trust in results, the infections captured in routine data may be incomplete or delayed, and not representative of infections being treated in the facility^28^. Similarly, in scenarios where sub-groups of a population do not use formal healthcare sources, these groups may be systematically under-represented in hospital data, and yet marginalised groups can carry the greatest burden of drug resistance^29^. The resulting gap between data and infection incidence in some high burden settings is probably one of the biggest challenges facing our AMR surveillance (Fig. 2).

Another key limitation of the routine data approach is that while it is generally feasible for large tertiary public hospital settings to have a microbiology laboratory on-site, smaller hospitals or facilities often buy microbiology services from a central facility, in a ‘hub-and-spoke’ model. While recent policy reports have laid out the cost savings of aggregating laboratories, other settings, such as smaller hospitals, primary care or community clinics, or private healthcare providers, face longer turnaround times shuttling samples to clinics, and more distant ‘service provider’ relationships with off-site microbiologists. Indeed, the consolidation of laboratory services in England led to far fewer cost-savings than initially predicted and, in the US, to long turnaround times. Importantly, it appears that off-siting non-patient facing services such as laboratories can make them vulnerable to creeping privatisation in universal public healthcare systems^30^. In principle, centralization of microbiology services could potentially be beneficial for surveillance as the data is already standardized across facilities; however it can also introduce complexities around data governance, ownership, linkage, and sharing as typically the individual ‘spoke’ facilities have the responsibility for surveillance (both facility-level and reporting externally) and linkage to their local EHR data.

The strategies and tools outlined above focus on the use of routine hospital data, however the vast majority^31^ of infections are managed outside of hospitals, in primary care settings where the vast majority of antibiotics are prescribed (e.g. 70% in the UK^32^). In principle, routine clinical data gathered in these settings could also be used for AMR surveillance and many of the issues outlined above, such as the need for linked and standardised data, equally apply outside hospital settings though often for less clinically severe cases. The importance of strengthening AMR data streams outside the hospital was recently emphasised by the 2024 Trinity Challenge on AMR, a >£2 M prize fund established to support innovation in “new capabilities and tools for collecting and using data from community settings” that has announced support for four projects in this area.

Other sources of AMR surveillance data include structured point prevalence surveys^33–37^ and research studies, including clinical trials or observational studies including those modelled on the ACORN (A Clinically-Oriented Antimicrobial Resistance Surveillance Network) protocol^38^. Research studies may specifically provide microbiological services that would not normally be available under routine clinical care (e.g. providing cultures and susceptibility testing free-of-charge for all patients meeting study inclusion criteria). Research data may thus yield clinical microbiology data on AMR that are more comprehensive than routine data. Still, research is time-limited and requires dedicated funding, so it often has a limited impact on building sustained capacity for better routine diagnostics^39^. Importantly, research data are designed to be representative of a defined study population, which may be only a subset of the general population (e.g. a research study might focus on particular age groups, patients with specific conditions or the most severe disease, or those admitted to particular wards) and therefore may be of limited use for AMR surveillance.

## Conclusions

To reduce AMR-associated mortality and morbidity, a key strategic priority^25^ is strengthening clinical microbiology, including diagnostics and susceptibility testing^11^. This is arguably most important at the hospital level where, typically, the most severe infections are diagnosed and treated, with the central goals being to improve the number of patients receiving timely administration of effective life-saving antibiotics (through diagnostics and empirical therapy), and to reduce the risk of healthcare-associated infections in patients admitted for other reasons (through infection prevention and control, or IPC). Whilst repurposing this clinical microbiology data for AMR surveillance has high value for the public health sector, it is important to recognise that public health priorities differ from those of hospitals and clinical microbiology labs. Therefore, for AMR data collection to be sustained for the benefit of both clinical and public health, it needs to benefit the hospital first, and public health second. Infrastructure and tools that support not only data collection, but timely data aggregation and use at the facility level, should therefore be prioritised over those geared toward data capture and onward reporting primarily for surveillance.

Whilst routine clinical data has public health value for AMR surveillance, this can only be realised effectively if specific resources are in place to facilitate this. In addition to human resources, there is a need to develop data standards for reporting microbiology laboratory data; and standards and tools for linking laboratory, clinical, and pharmacy data. The free and mostly open source tools outlined above go a long way towards providing the necessary resources, and would be further strengthened by the development of agreed data standards and formats for reporting microbiology data, with linked treatment and outcome data, to improve interoperability between tools and reporting streams.

It is also important to recognise that routine data is just one source of AMR surveillance. Other components include point prevalence surveys and ward-focused syndromic surveillance (such as the ACORN protocol^38^); these approaches too would benefit from strengthening of laboratory capacity, both at the facility level and more centrally in a hub-and-spoke model.

Finally, the ultimate value of AMR surveillance data will be determined by its accessibility to a wide range of users. This includes not only clinicians, healthcare facility managers, and multiple layers of the public health system, but also users outside of health systems where it can be used creatively to address different research questions, support innovation, and prioritise product development. The 2024 Political Declaration on AMR^1^ recognises the need for research and development across a wide range of areas, including innovation in diagnostics, vaccines, and therapeutics, all of which need AMR surveillance data to guide the prioritisation of target pathogens, drugs and/or syndromes. Therefore, data-sharing solutions must address not just the practicalities of sharing within health systems, but the potential privacy, ethical and legal concerns so that data derived from routine clinical care can be shared as widely as possible to maximise public benefit. Developing technical solutions, including automated de-identification and data aggregation that preserve individual privacy while maintaining maximum transmissible information content, should therefore be prioritised.

## Data Availability

No datasets were generated or analysed during the current study.
